# Effect of ZnO Nanoparticles Coating Layers on Top of ZnO Nanowires for Morphological, Optical, and Photovoltaic Properties of Dye-Sensitized Solar Cells

**DOI:** 10.3390/mi10120819

**Published:** 2019-11-26

**Authors:** Muhammad Saleem, W. A. Farooq, M. I. Khan, Majid. Niaz. Akhtar, Saif Ur Rehman, Naseeb Ahmad, Muhammad Khalid, M. Atif, Mona A. AlMutairi, Muhammad Irfan

**Affiliations:** 1 Department of Physics, Khwaja Freed University of Engineering and Information Technology, Rahim Yar Khan 64200, Pakistan; saleem.malikape@gmail.com (M.S.); ptckhan3@gmail.com (N.A.); 2 Department of Physics and Astronomy, College of Science, King Saud University, Riyadh 11451, Saudi Arabia; atifphy@gmail.com (M.A.); monaalmutairi@gmail.com (M.A.A.); 3 Department of Physics, The University of Lahore, Lahore 53700, Pakistan; iftikharphysicsuet@gmail.com; 4 Department of Physics, Muhammad Nawaz Sharif (MNS) University of Engineering and Technology, Multan 60000, Pakistan; 5 Department of Physics, COMSATS University Islamabad, Lahore 54000, Pakistan; saifuetian@gmail.com; 6 Department of Chemistry, Khwaja Fareed University of Engineering and Information Technology, Rahim Yar Khan 64200, Pakistan; muhammad.khalid@kfueit.edu.pk; 7 CAS Key Laboratory of Strongly Coupled Quantum Matter Physics, USTC, Hefei 230026, China; awazirzada@ksu.edu.sa

**Keywords:** DSSCs, coating layers of NPs, nanowires

## Abstract

This paper reports on the synthesis of ZnO nanowires (NWs), as well asthe compound nanostructures of nanoparticles (NPs) and nanowires (NWs+NPs) with different coating layers of NPs on the top of NWs and their integration in dye-sensitized solar cells (DSSCs). In compound nanostructures, NWs offer direct electrical pathways for fast electron transfer, and the NPs of ZnOdispread and fill the interstices between the NWs of ZnO, offering a huge surface area for enough dye anchoring and promoting light harvesting. A significant photocurrent density of 2.64 mA/cm^2^ and energy conversion efficiency of 1.43% was obtained with NWs-based DSSCs. The total solar-to-electric energy conversion efficiency of the NWs+a single layer of NPs was found to be 2.28%, with a short-circuit photocurrent density (*J*_SC_) of 3.02 mA/cm^2^, open-circuit voltage (*V*_OC_) of 0.74 V, and a fill factor (FF) of 0.76, which is 60% higher than that of NWs cells and over 165% higher than NWs+a triple layer of NPs-based DSSCs. The improved performance was obtained due to the increased specific surface area for higher dye anchoring and light harvesting of compound nanostructures with NWs+a single layer of NPs.

## 1. Introduction

Dye-sensitized solar cells (DSSCs) are considered promising devices for energy harvesting and large-scale fabrication due to their relatively high efficiency, costeffectiveness, and simple preparation process [1,2]. Many types of photovoltaic devices have been inducted in the market during the last 50 years [3,4,5]. However, their large-scale utility is still limited due to some challenges related to their stability, durability, the number of interparticle hops, and the low surface area [6,7,8]. So for, many ZnO photoanode architectures such as nanowires [9,10,11], nanobelts [12,13], nanoparticles [14,15,16], and nanotubes [10,17,18] have been fabricated for DSSCs. Recently, one-dimensional (1D) ZnO nanostructures have been extensively studied, which have the capacity to provide a direct pathway and less trapping sites than the random network for the fast collection of photogenerated electrons, and thereby reduce the number of interparticle hops [19,20,21,22,23]. However, such 1D nanostructures seem to have insufficient surface area, which constrains solar-to-electric energy conversion efficiency to a relatively low level compared to TiO_2_ nanostructures. Therefore, one way to further boost the efficiency of a cell is to pull off the light-harvesting ability of the photoelectrode film. One effective way to overcome these problems is either by utilizing optical enhancement effects, which can be achieved by mixing NPs with ZnO NWs, or by using a nanospheres film instead of a nanoparticles film. In several studies, different fractions (mass ratio) of NPs of ZnO/TiO_2_ were mixed with ZnO/TiO_2_ nanowires, nanorods, and nanofibers for affording a rapid electron conduction pathway and enlarging a dye adsorption area for increasing cell performance [24,25,26,27,28,29]. However, for significant enhancement in the overall performance of the cell, we still need an increase in the surface area of nanowire arrays [30].

Herein, we have made the use of ZnO nanowires (NWs) and compound nanostructures of ZnO nanowires and nanoparticles (NWs + NPs) with different coating layers of NPs on top of NWs as the photoelectrodes in DSSCs. The key point of this study is the coating of single, double, and triple layers of NPs and to study the effect of the coating layers’ thickness on the morphological, optical and photovoltaic properties of DSSCs. To enhance the performance of ZnO NWs DSSCs, nanoparticles of ZnO were filled in the interstices of NWs to achieve a fast electron transport pathway and to enlarge the dye adsorption area. Surprisingly, it is noted that DSSCs fabricated with double and triple layer coatingsof NPs show worse performance than those of NWs/NWs with a single layer of NPs. This can be due to the increased thickness, as an overly thick film aggravates unnecessary charge recombination and poses more constraint on mass transfer. Thus, we can infer that the appropriate content of NP incorporation into the NWs can promote the dye adsorption, transport the electrons, decrease the charge recombination, and thereby increase the performance of the cells.

## 2. Experimental

### 2.1. Synthesis of ZnO Nanoparticles 

**Figure 1:**
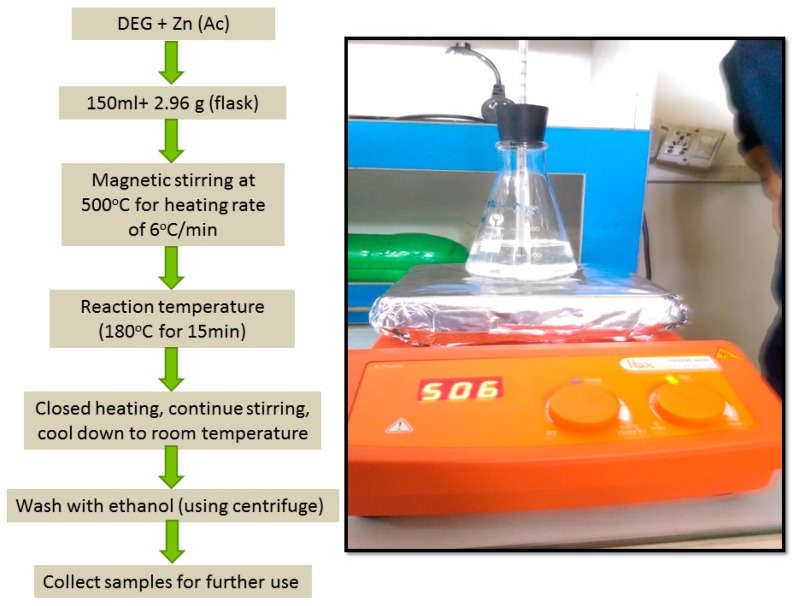
Procedure of the experiment.

NPs of ZnO were synthesized by dissolving 2.96 g of zinc acetate dihydrate (Zn (CH_3_COO)_2_.2H_2_O, Merck, Kenilworth, NJ, USA) (ZAD) in 150 mL of diethylene glycol (C_4_H_10_O_3_, Merck, Kenilworth, NJ, USA) (DEG) in a conical flask. The synthesis was performed in the following steps:(i) prepare the solution, (ii) heat it for hydrolysis, and (iii) heat treatment of the final samples. The experimental flask was fitted with a thermometer (Figure 1) and placed on hot stirrer, under continuous stirring. The heating of solution was carried out to 500 °C at a rate of 6 °C/min. When the temperature of the clear solution reached 180 °C, the solution first turned yellow and then into a milky white color. The temperature of the solution was fixed at 180 °C for 15 min and then stopped heating. When the reaction stopped and the temperature of the solution reached 26 °C, the white precipitate was collected by centrifuge and washed three times with ethanol. The powder was placed in a furnace at 400 °C for 1h to remove extra residual organics.

### 2.2. ZnO Nanowire Arraysand Nanoparticle Compound Structures Synthesis 

**Figure 2:**
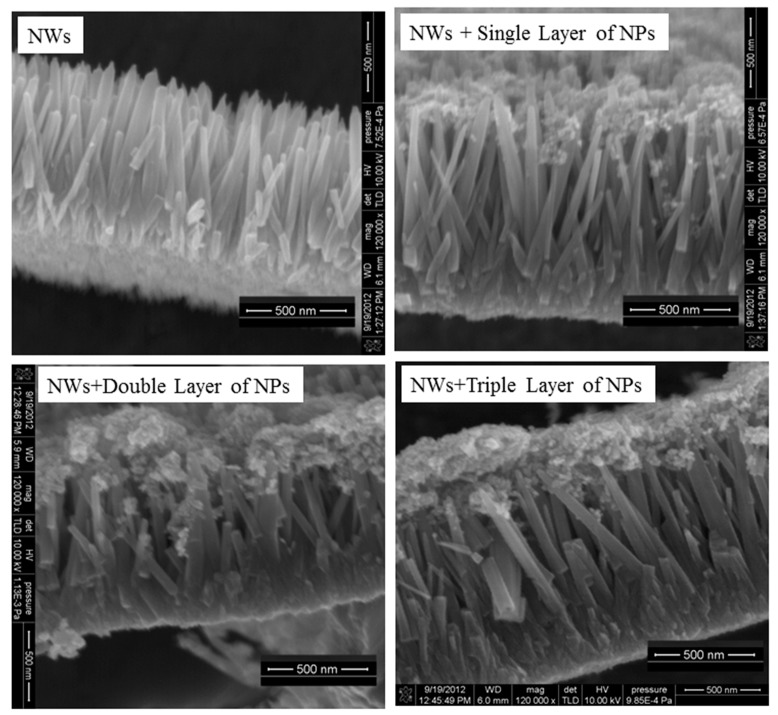
SEM images of the ZnO nanoproducts.

ZnO NWs arrays were synthesized in an aqueous solution, using a two-step process described elsewhere [31]. NWs were grown by immersing the seeded FTO substrates vertically in glass bottles containing 55 mL of solution. The growth solution consists of 1.09 g of zinc nitrate hexahydrate (ZNH), 0.70 g hexamethylenetetramine (HMTA), and 0.4 M ammonium hydroxide (NH_4_OH). Then, the bottles were covered and placed in an electric oven at 95 °C for 4 h. After growth, the substrates were immediately washed with distilled water. Finally, the NWs grown on substrates were backed at 450 °C for 30 min to remove excess solvent. The samples were prepared using spincoating. The concentration of ZnO nanoparticles synthesized in DEG at 500 °C at a rate of 6 °C/min) was 0.2 g in 30 mL of acetone. One sample consist only nanowires (NWs), the second sample was composed of nanowires and a single layer of nanoparticles (NWs+single layer of NPs), the third sample was composed of NWs and a double layer of NPs(NWs+double layer of NPs), and the fourth sample was composed of NWs and three layers of NPs (NWs+triple layer of NPs), as shown in Figure 2.The speed of the spin coater for the grown ZnO NPs onto NWs was 2500 rpm for 30 s. After each spincoating, the films were placed into a furnace for heating at 200 °C for 10 min to evaporate the solvent. This procedure of spinning to preheating was repeated three times for the sample having a triple layer of NWs+NPs.

### 2.3. Measurements and Characterizations

XRD patterns were measured by X-ray diffraction (XRD, Bruker D2 Phaser, Beerlika, MA, USA) using a 2θ scan. A scanning electron microscope study was done by SEM (model: JEOL JSM-7600F, Tokyo, Japan). I–V measurements of DSSCs were taken out with a Keithley 2400 source meter under an AM 1.5 global air mass filter, and the voltage sweep rate was 0.01V/s. The optical properties after dye loading were examined using a Perkenelmer Lambda-750 UV-VIS NIR spectrophotometer.

### 2.4. Cell Assembly

The effective area of photoelectrodes was 0.5 cm × 0.5 cm = 0.25 cm^2^. Dyeing was carried out by dipping these films in solutions of N719 dye for 3 h, and the films were then removed from the dye solution. The dyed films were rinsed off with acetonitrile to remove an excess of N719 dye remaining on the surface of the films. The dye was prepared by 0.5 mMof di-tertbutylpyridylcis–bis“(4,4′-dicarboxy-2,2′-bipyridine)dithiocyanato ruthenium(II)”, in a 1:1 mixture of acetonitrile. After dyeing, DSSCs were assembled by placing Pt-coated (8 Ω·cm^−1^) counter electrodes on prepared photoelectrodes separated by 30 µm of hot-melt surlyn film. The iodide/tri-iodide electrolyte consisting of 0.05 M I_2_ and 0.5 M 1,2-dimethyl-3-propylimidazolium iodide in acetonitrile was filed between the space of each cell by capillary action. Finally, three cells of each sample were fabricated and tested to check the reproducibility and almost similar results of I–V measurement happened.

## 3. Results and Discussion

### 3.1. Structural Analysis

**Figure 3:**
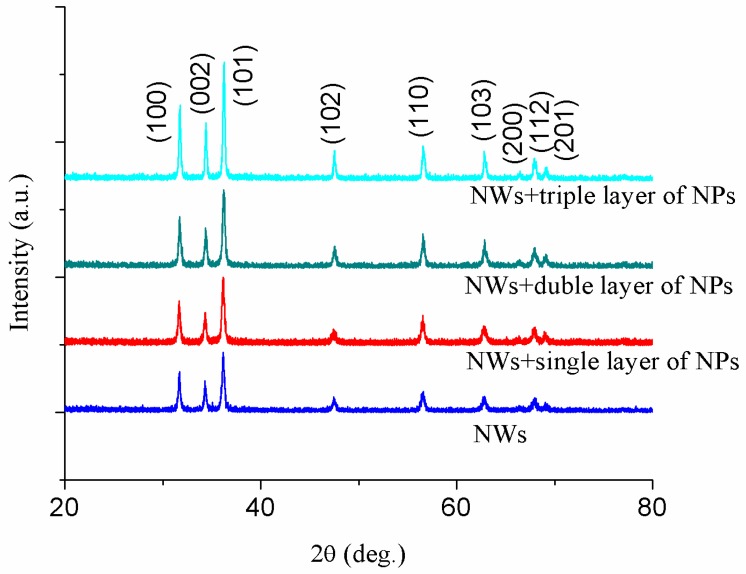
X-ray diffraction(XRD) patterns of the ZnO nanoproducts.

Figure 3 depicts the crystalline orientation of the ZnO NWs, NWs+a single layer of NPs, NWs+a double layer of NPs, and NWs+a triple layer of NPs compound structures determined by XRD. The XRD peaks of all the structures correspond to 2θ=31.7° (100), 34.4° (002), 36.5° (101), 47.7° (102), 56.8° (110), 63.1° (103), 66.5° (200), 68.1° (112), 69.2° (201) planes of ZnO. The XRD data also show the wurtzite structure of ZnO. These planes agreed well with the standard reported values (JCPDS file No. 36-1451). There are no diffraction peaks corresponding to impurities such as Zn and Zn (OH)_2_.The crystallite size of nanoparticles was calculated for the (101) peak due to high intensity using the Scherrer equation [31]. The calculated size of nanocrystallites was 20 nm. The intensity of peaks increases with the increase in thickness due to the growth of NP layers. All the peaks are slightly shifted toward right with the addition of NPs and the absence of any other peak confirms the high purity of the hexagonal ZnO phase and the good alignment of the samples. It is noteworthy that the intensity of (101) is strongest among the other reflections.

### 3.2. Morphological Analysis

**Figure 4:**
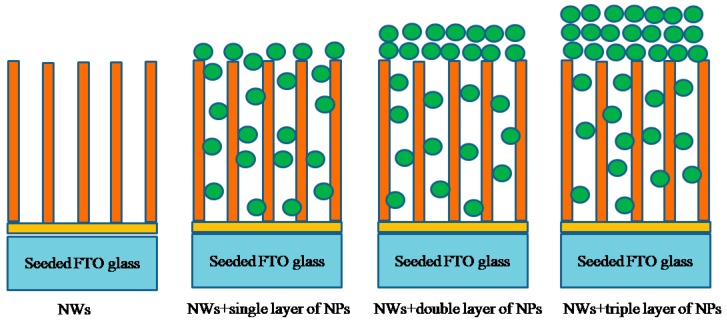
Schematic representation of photoelectrodes made from nanowires (NWs), NWs+a single layer of nanoparticles(NPs), NWs+a double layer of NPs, and NWs+ atriple layer of NPs.

**Figure 5:**
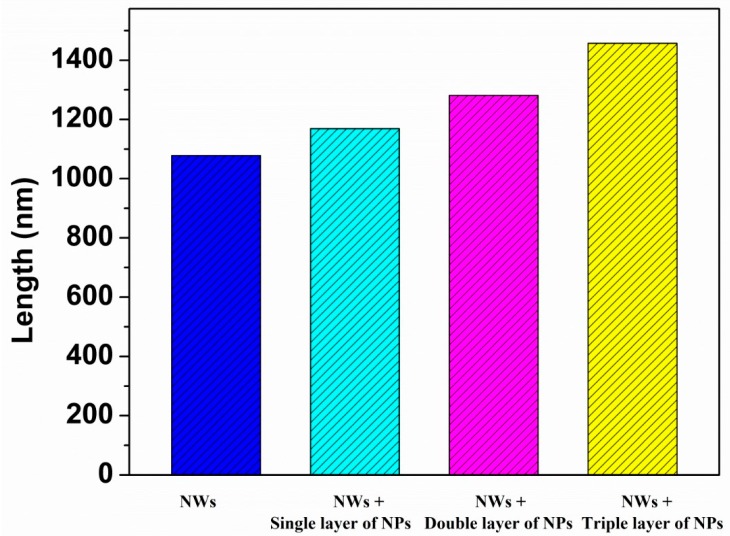
Histogram of increase in length (thickness) with different coating layers.

The crystallographic morphologies of the NWs of ZnO, NWs+a single layer of NPs, NWs+a double layer of NPs, and NWs+a triple layer of NPs were observed using FESEM. From Figure 2, it can be seen that the alignment is good, and the NWs are vertically grown on the substrates (FTO) with a length of about 1.1 μm. As a result, such a type of one-dimensional nanostructured-based photoelectrode film has insufficient surface area. If the interstitial voids between the NWs might be filled with NPs, the photoelectrodes’ surface area would be increased. To overcome this deficiency of surface area, we have spin-coated ZnO NPs on top of NWs. For a single layer of NPs (Figure 2), besides the formation of a topping layer, it can be seen that nearly all of the NPs fill the interstices between the NWs, yielding a combined structure of NWs and NPs. In case of double layers, NPs were detached on top of the NWs. Some NPs were partially penetrated and dispersed between the gaps of nanowires, but a large portion of NPs remained coated on the surface of the nanowire arrays. However, in case of triple layers, NPs were sitting on the top of the nanowire arrays without any infiltration between the gaps of NWs except for those that dispersed during the first and second layer. As a result of this infiltration, a thick layer of NPs is built on the surface of NWs (Figure 4, Schematic), which reduces the penetration (diffusion) of electrons from NPs to NWs and increases the trapping/detrapping events. The average thickness of the nanoparticles coating measured by Image J software (Figure 5, Histogram) was 90, 172, and 275 nm for the single, double, and triple layers, respectively.

### 3.3. Optical Analysis of Dye/ZnO Anodes

**Figure 6:**
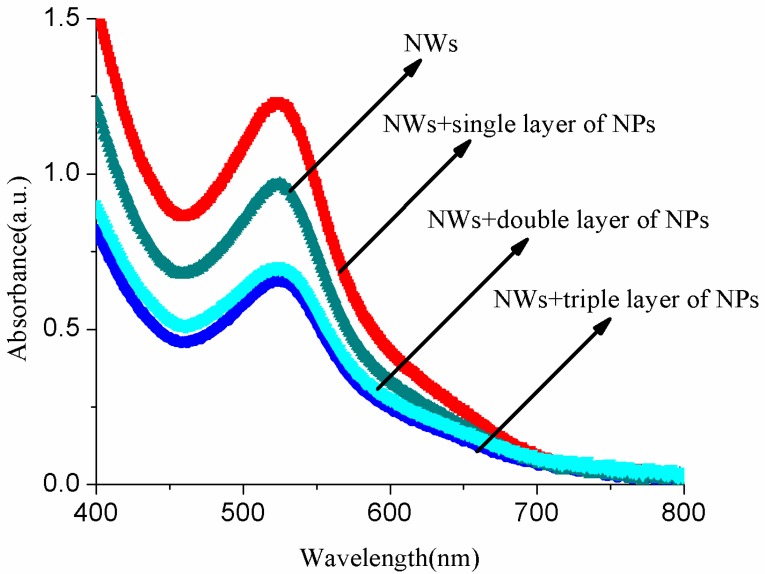
Optical absorbance of N719 dye adsorbed on ZnO compound nanstructures.

In Figure 6, the absorption spectra of dye/ZnO NWs, NWs+a single layer of NPs, NWs+a double layer of NPs, and NWs+a triple layer of NPs are shown. It is observed that all the NWs and NWs+NPs electrodes present an absorption peak at 520 nm. These peaks at 520 nm in all the electrodes are due to a visible 4d-π* metal-to-ligand charge transfer (MLCT) in N719 dye [32]. Due to the presence of coupling strength between the surface of ZnO NWs and NWs+NPs electrodes and excited dye, these absorption peaks are slightly blue shifted. The different NPs layers on the top of NWs will provide a large surface area for dye loading, and hence improve the light harvesting capability of photoelectrodes. It is clear from the absorption spectra (Figure 6) that the dye adsorption capacity of NWs and NWs+NPs films with different layers of NPs is rated in the following order, from high to low: the single layer of a NP compound nanostructure (NWs+a single layer of NPs), nanowires (NWs), a double layer of a NP compound structure (NWs+a double layer of NPs), and a triple layer of a NP compound structure (NWs+a triple layer of NPs). It is evident from the spectra that the single layer of a NP (NWs+a single layer of NPs) compound nanostructure has the highest dye adsorption capacity, because such a configuration contributes more surface area for dye molecules. Hence, as a result, the current density (*J*_SC_) and overall light-to-energy conversion efficiency would increase. The dye adsorption capacity of NWs is less than NWs+a single layer of NPs due to the lower surface area. The other two samples, i.e., NWs+a double layer of NPs and NWs+a triple layer of NPs, show lesser dye adsorption capacity because of the presence of double and triple layers of NPs on the top of the NWs. Since the dye adsorption becomes weaker as the thickness of the layers increases, a thicker layer blocks the complete infiltration of dye into the whole sample. Chang et al. and other researchers proposed that insufficient film thickness leads to a low interfacial surface area, whereas an overly thick film aggravates unnecessary charge recombination and poses more constraint on mass transfer [33,34,35,36]. As a result, a photoanode that is too thin or too thick results in lower conversion efficiency. Furthermore, it also causes higher electron transport resistance and increases the recombination of electrons with $I_{3}^{-}$ on the ZnO surface, resulting in smaller open circuit voltage and efficiency.

### 3.4. Current Voltage (I–V) Analysis of DSSCs

**Figure 7:**
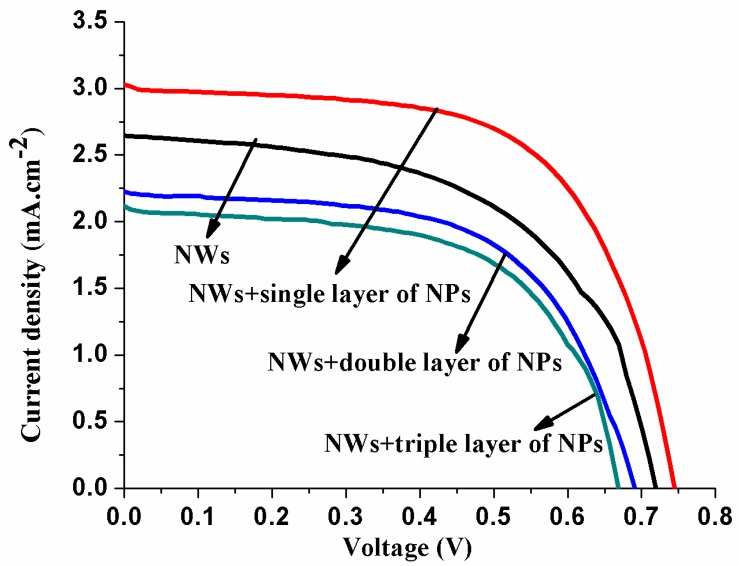
*J*–*V* characteristics for ZnO compound nanostructures.

Figure 7 shows the photoelectric characteristics for NWs, NWs+a single layer of NPs, NWs+a double layer of NPs, and NWs+a triple layer of NP compound structure-based solar cells. The parameters measured from I–V curves for thebest cells prepared from different coating layers of NPs on ZnO nanowires are listed in Table 1. Under illumination, the NWs cell show a*J*_SC_ of 2.64 mA/cm^2^, fill factor (FF) of 0.74, *V*_OC_ of 0.72 V, and *η* of 1.43 %.The NWs photoelectrodes have insufficient internal surface area that results in lower dye loading and lightharvesting [20,21,37,38]. The NWs+a single layer of NPs compound structure shows a*J*_SC_of 3.02 mA/cm^2^, FF of 0.76, *V*_OC_of 0.74 V, and *η*of 2.28%. The *J*_SC_, *η*, and overall performance obtained from the NWs+a single layer of NPs-based DSSC are substantially increased compared with those of NWs cells alone. The significant enhancement in these results was possibly caused by the superior lightharvesting of the NWs+a single layer of NPs DSSC, in which NPs provide the higher specific surface area for more dye anchoring and the NWs provide an effective pathway for rapid electron transport [27,28,39]. These results from the NWs and NWs+a single layer of NPs DSSCs closely matched with the results obtained from NWs, NRs, and nanotips [21,24,28,29,40,41,42], and the results of NWs+a single layer of NPs compound nanostructure show a considerable agreement with the results reported by other researchers listed in Table 2 for comparison [27,43,44,45,46,47,48]. On the other hand, the other samples of NWs+a double layer of NPs and NWs+a triple layer of NPs show worse performance; i.e., *J*_SC_ decreases from 3.02 to 2.11 mA/cm^2^ and *η* decreases from 2.28% to 0.86 % (Figure 7, Table 1) compared withthose of NWs+a single layer of NPs. This is due to the double and triple layer of nanoparticles on the top of nanowires, because most of the nanoparticles are sitting on the top of nanowireswithout any infiltration, where the resulting nanoparticle–nanowire contact area is minimized. It is worth noting that nanoparticle–nanowire and nanoparticle–nanoparticle contact areas are also important [27]. From Figure 4, the interface between two phases can be seen clearly, and the nanoparticles do not fill the interstitial voids after coating double and triple layers. The morphology obtained from double and triple coating layers—the thick layers of nanoparticles—forms a bottleneck to electron transport, because all the current from a thick layer of nanoparticles must pass through the nanowires to reach the electrode. Moreover, this thick layer also causes higher electron transport resistance and increases the recombination of electron with $I_{3}^{-}$ on the ZnO surface, resulting in smaller *V*_oc_ and *η* values. The cells fabricated from double and triple layers of NP photoelectrodes have shown low efficiency compared with their NWs/NWs+a single layer of NPs-based DSSCs counterparts. This decrease in η is due to the following reasons.First, electron transfer from NP thick-layer electrodes is proposed to occur by a series of hopping events, which shows charge recombination and a non-exponential current, thereby slowing down excellent charge transport [31]. Second, the transport of electrons in the NPs film undergoes multiple trapping and detrapping processes. In addition, grain boundaries and mid gap states increased because of the thicker layers of NPs on the top of NWs, which results in a slow electron transport rate [49]. Third, an obvious drawback of the use of a double or triple layer of NPs on the top of NWs is that the thickness increases, whereas an overly thick film aggravates unwanted charge recombination and poses more constraints on mass transfer [33,34]. Thus, from the above discussion, we can infer that the appropriate content of NPs incorporation onto the NWs can promote the dye adsorption, transport the electrons, decrease the recombination of charge, and thereby increase the cells’ performance.

## 4. Conclusions

In summary, ZnO NWs and compound nanostructures of ZnO NWs+NPs with different coating layers of NPs on top of NWs were fabricated for the application of DSSCs. The transportation of electrons is high in compound nanostructures (NWs + NPs), and ZnO NPs filling the gaps between the ZnO NWs offers a high internal surface area for loading the dye and lightharvesting. The overall maximum efficiency of 2.28% for NWs+a single layer of NPs-based DSSCs was achieved with FF, *V*_OC_, *J*_SC_ values of 0.76, 0.74V, and 3.02 mA/cm^2^, respectively, which is 165% higher than that of their other counterparts. The improved performance was obtained due to the large surface area and light harvesting of compound nanostructures.

## Figures and Tables

**Table 1:** Parameters of ZnO nanostructured-based dye-sensitized solar cells (DSSCs).

Samples	Short-Circuit Photocurrent Density *J*_sc_ (mA/cm^2^)	Open-Circuit Voltage *V*_oc_ (V)	Fill Factor (FF)	*η* (%)
NWs	2.64 ± 0.021	0.72 ± 0.020	0.74 ± 0.025	1.43 ± 0.015
NWs + single of NPs	3.02 ± 0.010	0.74 ± 0.015	0.76 ± 0.020	2.28 ± 0.011
NWs + double of NPs	2.22 ± 0.005	0.69 ± 0.012	0.62 ± 0.005	0.93 ± 0.017
NWs + triple of NPs	2.11 ± 0.011	0.68 ± 0.010	0.58 ± 0.005	0.86 ± 0.001

**Table 2:** Comparison of performance parameters of DSSCs.

Samples	Short-Circuit Photocurrent Density *J*_sc_ (mA/cm^2^)	Open-Circuit Voltage *V*_oc_ (V)	Fill Factor (FF)	*η* (%)
ZnO NW–NP hybrid cell [27]	3.00	0.77	0.65	1.3
ZnO NW [44]	2.5	0.44	0.47	0.45
ZnO NW–NP composite [44]	8.33	0.58	0.58	2.77
ZnO NW [45]	4.55	0.60	0.41	1.16
ZnO NW–NP hybrid cell [45]	15.16	0.61	0.46	4.24
ZnO NW [46]	2.52	0.53	0.37	0.49
ZnO NW–NP hierarchical [46]	5.4	0.62	0.60	2.03
ZnO NR–NP hybrid cell [47]	5.39	0.52	0.57	1.6
ZnO NR–NP hybrid cell [48]	4.57	0.45	0.34	0.69
